# Navigating the risks and rewards of scavenging in multipredator, human‐impacted landscapes

**DOI:** 10.1002/ecy.70090

**Published:** 2025-05-08

**Authors:** Calum X. Cunningham, Rebecca Windell, Lauren C. Satterfield, Aaron J. Wirsing, Thomas M. Newsome, Taylor R. Ganz, Laura R. Prugh

**Affiliations:** ^1^ School of Environmental and Forest Sciences University of Washington Seattle Washington USA; ^2^ School of Natural Sciences University of Tasmania Hobart Tasmania Australia; ^3^ School of Life and Environmental Science University of Sydney Sydney New South Wales Australia

**Keywords:** anthropogenic landscape, carcass, mesocarnivores, mesoscavenger, risk–reward trade‐off, scavenging

## Abstract

Large carnivores can influence smaller scavengers through both positive and negative interactions (e.g., carrion provisioning and intraguild killing) and ultimately shape scavenging efficiency. However, we know little about this trade‐off in anthropogenic landscapes where humans kill carnivores and provide carrion subsidies. In the context of wolf (*Canis lupus*) recolonization of human‐impacted landscapes in Washington, USA, we investigated how sources of ungulate mortality (wolves, cougars [*Puma concolor*], and vehicles) shape scavenging efficiency, community‐wide carcass visitations, and the strategies used by scavengers to navigate risk–reward trade‐offs. Cougar and wolf kills mostly occurred in areas with low‐to‐moderate human influence, whereas roadkill typically occurred in areas with high human impact. Wolves consumed their kills most rapidly (median <4.7 days), providing fewer scavenging opportunities than cougar‐ and vehicle‐killed ungulates, which persisted longer (median = 8.9 and 12 days, respectively). Roadkill primarily attracted avian scavengers, whereas mammalian scavengers used roadkill to a lesser degree and did so by shifting to more nocturnal foraging. The absence in winter of turkey vultures (*Cathartes aura*) and black bears (*Ursus americanus*), which are obligate and apex scavengers, respectively, coincided with a seasonal increase in scavenging by most other species. The two mammalian mesocarnivores exhibited divergent strategies: Coyotes (*Canis latrans*) frequently scavenged but usually for short durations and with heightened vigilance at predator kills, whereas bobcats (*Lynx rufus*) visited carcasses less frequently but fed for longer durations and displayed low vigilance while scavenging. These results suggest a hierarchical decision‐making process whereby scavengers first choose whether to forage at a carcass before fine‐tuning foraging duration, using temporal refugia, or increasing vigilance. Predator recovery in human‐dominated landscapes therefore adds complexity to the spatiotemporal landscape of risks and rewards, and outcomes for scavengers will likely depend on their propensity to scavenge and vulnerability to humans and large predators.

## INTRODUCTION

Scavengers play crucial roles in efficiently cycling nutrients and reducing reservoirs for disease (Wilson & Wolkovich, 2011). However, scavenger richness is typically lower in human‐modified landscapes (Sebastián‐González et al., 2019), and several apex and obligate scavengers have suffered severe population declines, leading to impaired scavenging efficiency and the release of less‐efficient mid‐sized “mesoscavengers” (Cunningham et al., 2018; Fielding et al., 2022; O'Bryan et al., 2019). In some cases, though, species that scavenge, like gray wolves (*Canis lupus*), brown bears (*Ursus arctos*), and Eurasian lynx (*Lynx lynx*), are returning to parts of their former ranges (Chapron et al., 2014; Ripple et al., 2022). These recoveries could not only pose a risk to some smaller scavengers (Newsome et al., 2017; Prugh et al., 2009; Ritchie & Johnson, 2009), but they might also provide benefits by provisioning carrion (Elbroch & Wittmer, 2012; Wilmers, Crabtree, et al., 2003). Cougar (*Puma concolor*) kill sites, for example, attracted coyotes (*Canis latrans*) and provided substantial food subsidies in Oregon, USA, but cougars were also the main cause of coyote mortality (Ruprecht et al., 2021). These positive and negative interactions establish a risk–reward trade‐off that could markedly influence the structure of carnivore communities (Prugh & Sivy, 2020) and, in turn, the efficiency of carrion cycling.

Most of our knowledge of interactions between larger and smaller carnivores, especially involving wolves, comes from systems with large‐bodied prey and limited human influence (e.g., Yellowstone or Denali National Parks; Klauder et al., 2021; Walker et al., 2018; Wilmers, Crabtree, et al., 2003). In such systems, wolves provision scavengers, such as corvids, with a steady supply of carrion (Walker et al., 2018; Wilmers, Crabtree, et al., 2003). However, such scavenging effects could differ substantially outside of protected areas where humans kill carnivores at high rates (Darimont et al., 2015; Kuijper et al., 2016). Human presence may induce shorter foraging bouts and earlier abandonment of carcasses (Smith et al., 2017), as well as fear‐induced cascades to lower trophic levels (Suraci et al., 2016, 2019). Such avoidance of humans by larger predators can “shield” other animals (Berger, 2007), but this shield is complicated by the fact that humans also kill mesocarnivores at very high rates (Prugh et al., 2023). Humans additionally provide carrion subsidies that are widely used by carnivores, such as carcasses from roadkill, hunting, and livestock (Newsome et al., 2015). Moreover, compared to predator‐killed carcasses, those provided by humans are often more clustered spatially and temporally (Fielding et al., 2021; Wilmers, Stahler, et al., 2003) and are less likely to be defended by a large predator. Collectively, the synergistic human effects of instilling fear in large predators, shielding but also killing mesocarnivores, and provisioning carrion, have the potential to shape scavenger communities and carrion removal rates.

**FIGURE 1:**
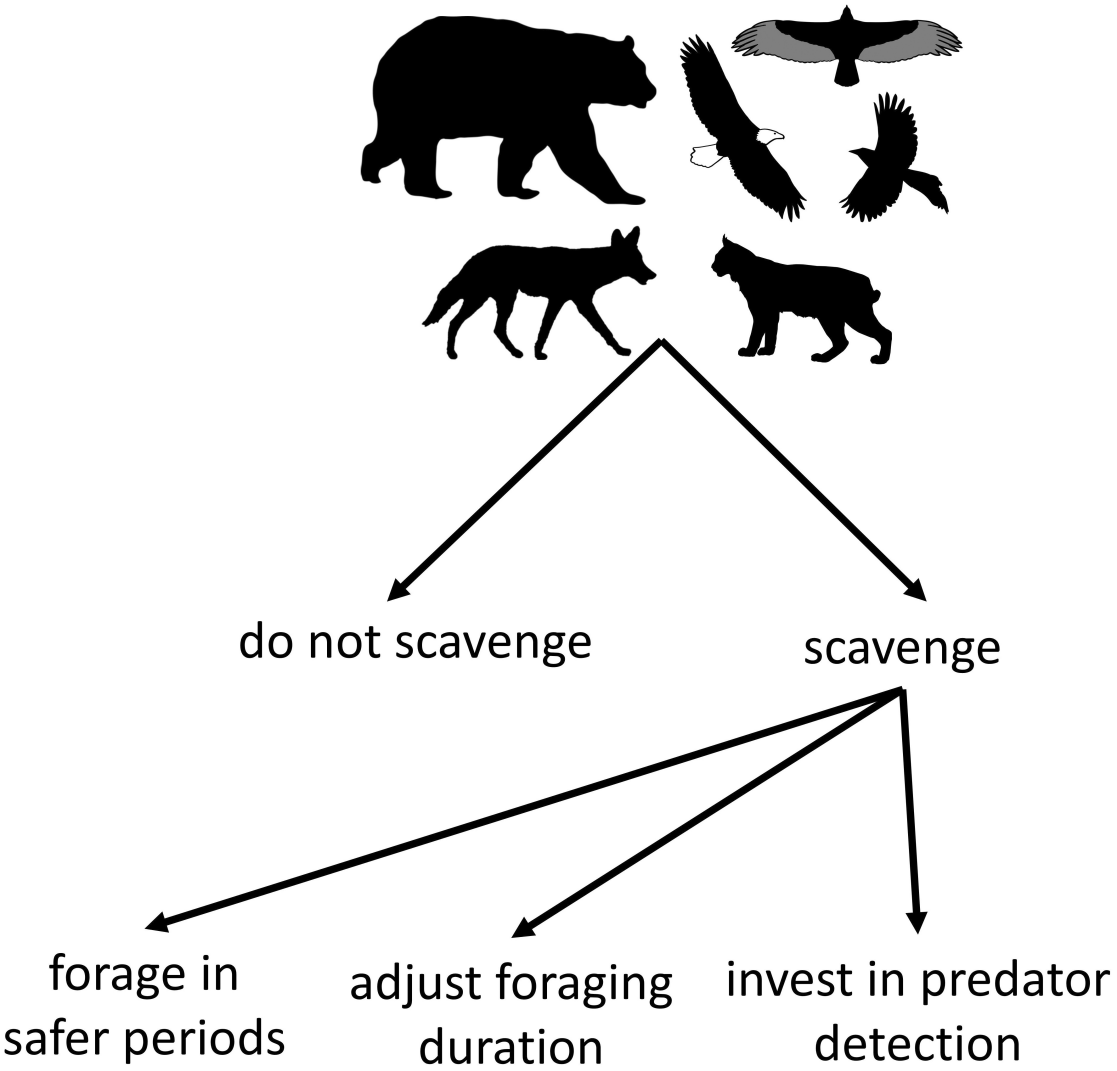
Conceptual model of the hierarchical decisions made by scavengers. Based on an animal's energetic needs and the perceived risk of exploiting a carcass, a scavenger must first decide whether to scavenge or not, after which it may attempt to reduce risk of injury or death through strategies such as finely adjusting foraging durations, foraging in temporal refugia, and use of vigilance. Each pathway corresponds with a separate analysis. Silhouette credits (PhyloPic; public domain under a CC0 1.0 Universal Public Domain Dedication license): black bears by Tracy Heath; bald eagle and turkey vulture by Andy Wilson; magpie by T. Michael Keesey; bobcat by Margot Michaud. Coyote silhouette by Bob Comix (Creazilla; public domain under a Creative Commons Attribution 4.0 license).

To manage the risk posed by larger predators (including humans), an optimally foraging scavenger should attempt to balance the benefits of feeding with the costs of injury or death (Brown et al., 1999). The specific strategies used to navigate this risk–reward trade‐off will vary among species (Ruprecht et al., 2021), presumably depending on factors such as a species' reliance on carrion and the predictability of risk (Palmer et al., 2022). Scavengers may choose to navigate risk through a hierarchical process (Figure 1), whereby they first decide whether to visit a carcass site (Klauder et al., 2021) before attempting to mitigate risk through strategies such as reducing time spent at risky carcasses, foraging in temporal refugia (sensu Smith et al., 2019), or increasing vigilance (Palmer et al., 2022). Because most studies of large carnivores have focused on protected areas or on a single large predator species, we have little empirical understanding of the effects of carnivore recovery on scavenging dynamics in general, or how scavengers negotiate the risk–reward trade‐off in multipredator, human‐dominated systems.

We hypothesize that scavenging dynamics are driven by fundamental trade‐offs between risks and rewards that are strongly influenced by anthropogenic factors outside of protected areas. Here, we evaluate this hypothesis by examining how sources of ungulate carrion influence scavenging efficiency, carcass visitations, and behavioral strategies used by scavengers in multiuse landscapes of Washington, USA, where wolf recolonization is ongoing. In 2008 after an absence of ~80 years, dispersing wolves from Idaho, USA, and British Columbia, Canada, established packs in northeastern Washington. By 2021, the population had increased to a minimum of 206 wolves from at least 33 packs (WDFW et al., 2022). Cougars were also intensely persecuted during the 1800s and early‐to‐mid 1900s (Gross, 2008), but they were never extirpated and are now widely distributed across the ~60% of Washington that is forested (Washington Department of Fish and Wildlife, 2022, p. 288). Gradients from forested public lands to highly modified agricultural and residential lands exert a strong influence on the distributions of Washington's wolf and cougar populations (Prugh et al., 2023), presenting scavengers with a complex, multispecies spatiotemporal landscape of risks and rewards (Palmer et al., 2022).

In this study, we monitored carrion from wolf kills, cougar kills, and vehicle collisions, which provide contrasting features that should affect the scavenger community. Wolves typically hunt as packs, with larger packs capable of killing larger animals (MacNulty et al., 2014) and perhaps consuming carcasses more rapidly than solitary predators. Cougars are usually solitary hunters with secretive feeding habits that involve caching carcasses beneath vegetation (Allen et al., 2023; Laundré & Hernández, 2003), which may reduce discovery rates by competitors. At a landscape scale, roadkill occurs in predictable locations (i.e., along roads), but use of roads by humans suggests roadkill may pose the highest risk for large carnivores, while offering the greatest opportunities to the wider scavenger community. We tested three primary predictions: (1) carrion persistence and provisioning should be higher from anthropogenic sources (i.e., roadkill) than from large carnivores (Prugh & Sivy, 2020); (2) seasonal dynamics should alter the risk–reward landscape because dominant (i.e., bears) or obligate (i.e., vultures) scavengers are present only during warmer months, allowing subordinate scavengers to make greater use of carrion in cooler periods; and (3) risk‐sensitive behaviors, such as temporal partitioning and vigilance, should vary among subordinate scavengers in response to the highest perceived threats (i.e., large carnivores on their kills and humans near roadkill). We use the results to infer how predator recovery in anthropogenic landscapes is likely to influence the broader scavenger community through interactions at carcasses and access to carrion.

## METHODS

### Study area and carnivore community

**FIGURE 2:**
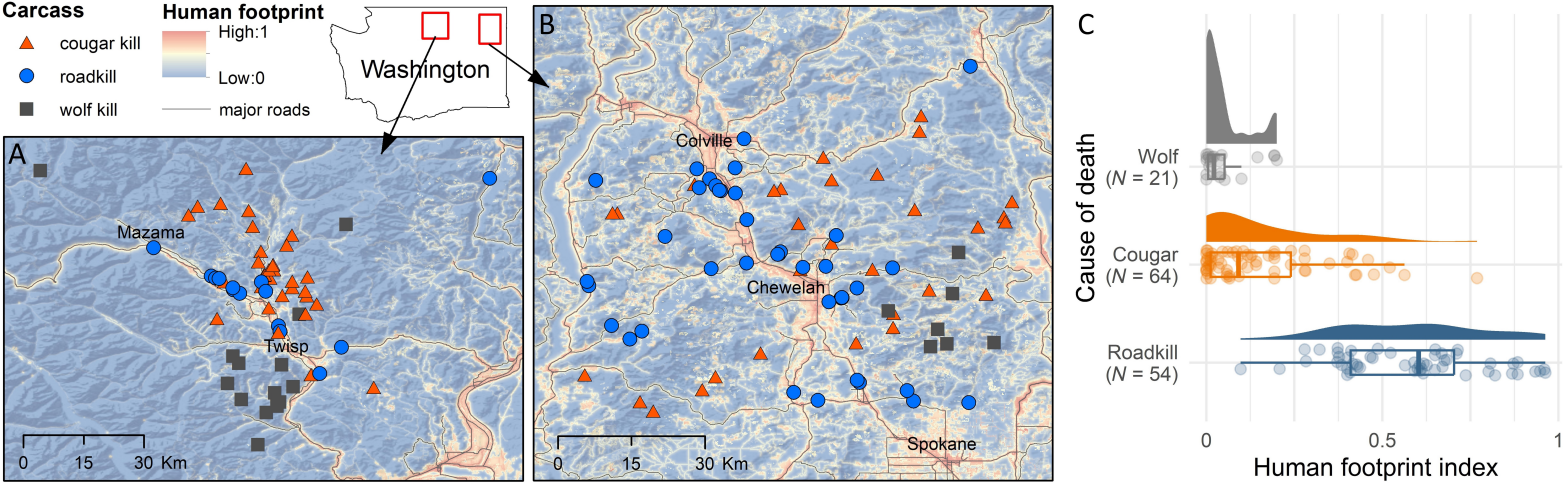
Distribution of carcasses differs according to cause of death and human influence. The study took place in two regions of Washington, the Okanogan (A) and northeastern Washington (B). (C) Box and density plots show that wolf and cougar kills usually occurred in areas of relatively low human footprint index, whereas roadkills were usually in areas of considerably higher human influence.

This study was conducted as part of the Washington Predator Prey Project—a collaborative study between the University of Washington and the Washington Department of Fish and Wildlife—that was established to explore the ecological effects of wolf recovery in Washington's multiple‐use landscapes. The study was conducted across two large study areas, Okanogan and Northeast, in northern Washington, USA, from 2017 to 2021 (Figure 2). Both were similar in size (~5000 km^2^) and consisted of a mix of private and public land, used mainly for timber and livestock production, agriculture, recreation, and residential development (Figure 2).

The focal species ranged from obligate to facultative scavengers. Turkey vultures (*Cathartes aura*) are obligate, migratory scavengers that can form large groups at carcasses and may exclude smaller avian scavengers (Prior & Weatherhead, 1991). Common facultative avian scavengers included common ravens (*Corvus corax*), black‐billed magpies (*Pica pica*), golden eagles (*Aquila chrysaetos*), and bald eagles (*Haliaeetus leucocephalus*). The largest mammalian scavenger, the black bear (*Ursus americanus*), has been documented monopolizing carcasses and kleptoparasiting cougar kills (Allen et al., 2014). Coyotes and bobcats were the most common mammalian mesoscavengers, with broadly similar site occupancy in this landscape (Bassing et al., 2023). Although coyotes and bobcats are both facultative scavengers, research in Oregon indicates that coyotes are substantially more inclined to scavenge than bobcats (Ruprecht et al., 2021). The obligate scavenger (turkey vulture) and the apex scavenger (black bear) are both only active in the region during the warmer months, as vultures migrate south for winter and black bears hibernate. Unlike other focal regions of wolf research where common prey are typically large (e.g., elk, *Cervus canadensis*, bison, *Bison bison*, and moose, *Alces alces* in Denali, Yellowstone, and Isle Royale National Parks; Peterson et al., 2014), the ungulate community in Washington is mostly composed of mule deer (*Odocoileus hemionus*) and white‐tailed deer (*Odocoileus virginianus*), with elk and moose present in much lower numbers (Bassing et al., 2023).

### Data collection

We deployed remote cameras (Reconyx Hyperfire [92%], Moultrie M‐880 [4%], or Bushnell Trophy Cam [4%]) to monitor ungulate carcasses and kill sites. We located carcasses in three ways: (1) investigating clusters of GPS‐collared wolf and cougar locations, (2) investigating mortalities of ungulates monitored with global positioning system (GPS) or very high frequency radio (VHF) collars, and (3) opportunistic sourcing of roadkill. Investigators of the Washington Predator Prey Project captured and collared 16 wolves (6 packs), 60 cougars, 149 mule deer, 273 white‐tailed deer, and 93 elk across the two study areas. At most times of the study, at least one member of each wolf pack was collared, and collared cougars were distributed widely across both study areas, with both species showing avoidance of areas heavily impacted by humans (Prugh et al., 2023). GPS collars were generally set to record one location every four hours, and this information was used to identify wolf kills, cougar kills, and mortalities of collared ungulates. Neonatal (age 0–10 days) white‐tailed deer and elk were fitted with VHF collars rather than GPS collars (Ganz et al., 2024). Wolves were collared as part of existing management activities by the Washington Department of Fish and Wildlife. Captures of the other species were carried out in accordance with the University of Washington Institutional Animal Care and Use Committee (IACUC) Protocol #4226‐01.

To identify wolf and cougar kills, field crews ran a cluster detection algorithm each morning to identify clusters of at least two GPS locations within 300 m of each other within a 72‐h time window (rASF R package; Mahoney & Young, 2017). We attempted to locate potential kill sites, determine the prey species, age class, sex, cause of death, and estimate percentage carcass biomass remaining. Estimated date of death was inferred from the onset of the GPS cluster. Cougar clusters were visited as soon as possible in winter and summer. Wolves were listed as Endangered at the state level (WDFW, 2019), and consequently, there were restrictions on visiting wolf clusters. From 2017 to 2019, visiting a wolf cluster was only permitted once four days had elapsed after wolves abandoned the cluster site. In 2020–2021, this restriction was amended to one day after abandonment by wolves. Cluster investigations of wolf kills were also restricted to winter months to avoid the potential of disturbing wolves at den or rendezvous sites. As such, the final dataset contained only one wolf kill in summer, limiting our ability to detect seasonal differences in scavenging patterns on wolf kills.

Ungulate (white‐tailed deer, mule deer, and elk) collars were equipped with mortality sensors that notified field crews when a collar was inactive for a period (9 h for adults, 8 h for elk calves, and 6 h for deer fawns). A mortality notification triggered a field search as soon as possible, which included an attempt to determine the cause of death and the estimated percentage carcass biomass remaining. Estimated date of death was inferred from the time‐stamped mortality notification and the onset of the GPS cluster. We included ungulate mortalities for analysis in this paper when the cause of death could confidently be assigned to wolves, cougars, or vehicle strikes. Cause of death was estimated in the field and confirmed by genetic analysis in the case of predator kills (for details, see Ganz et al., 2022). The median elapsed times from the onset of the cluster/kill to the investigation for cougar kills were 2.5 days (range = 0.8–26) and for wolf kills were 4.2 days (range = 2–10).

Roadkill ungulates were located opportunistically during fieldwork. We installed cameras to monitor roadkill carcasses that were fresh and largely un‐scavenged. To prevent road clean‐up crews from removing carcasses, carcasses were typically located early in the morning and then moved no more than 50 m from the side of the road to place a camera. Carcasses found along stretches of road abutting private lands were moved to the nearest public parcel. In doing this, carcasses were moved no more than 5 km and placed >1 km from human residences.

Cameras were set to take 3 photographs per trigger with a 1‐s break between triggers. All photographs were tagged to species level and verified by a second coder using Timelapse software from which we calculated the time spent at kill sites based on the number of minutes each day in which a species was observed at the kill site. Hereafter, we interpret this measure as an “index of scavenging activity,” rather than an absolute measure of scavenging duration, because we did not specifically record whether an animal was feeding. For the analysis of scavenging activity, vigilance behavior, and interspecific interactions, we did not include data beyond 30 days from the estimated death of the ungulate, as the vast majority of edible biomass had usually been consumed by this point and animal activity had largely subsided. Each camera location was assigned (1) a value of human footprint index that was specifically created for this region by the Cascadia Partner Forum (https://cascadiapartnerforum.org/terradapt); (2) the proportion of forest cover in a 250 m radius around each carcass, also using land cover data from Cascadia Partner Forum; and (3) elevation from the Shuttle Radar Topography Mission (Farr et al., 2007). These variables each have the potential to influence scavenger distributions, ability to discover carcasses (e.g., visual, olfactory), and behavior following carcass discovery (Perrig et al., 2023). However, because humans preferentially occupy lower elevations and strongly influence the distribution of large carnivores in our study regions (Prugh et al., 2023), the proportion of forest cover was the only variable that was not confounded with cause of death (Appendix S1: Figure S1) and was thus the only one suitable for inclusion in subsequent models. Data are archived publicly (Cunningham et al., 2025).

**FIGURE 3:**
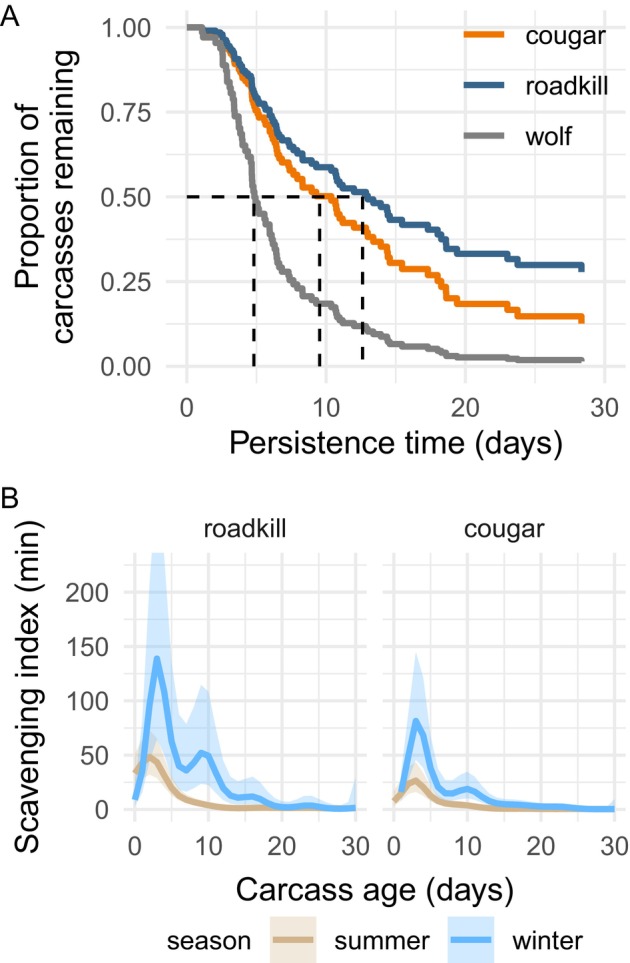
Differences in the persistence of carcasses and scavenging activity among causes of death and the seasons. (A) Colored lines show adjusted survival curves based on a Cox proportional‐hazards model, holding other covariates at their means. Dashed vertical lines show the model‐estimated median carcass persistence times. (B) Mean daily scavenging activity, summed across species, as carcasses aged. Lines show the fit (±95% CI) of a generalized additive mixed model. Scavenging activity was not analyzed on wolf kills owing to sparse observations of foraging.

### Statistical analysis

All analyses were conducted in R v4.2.2 (R Core Team, 2023). As recommended by Muff et al. (2021), we interpreted *p*‐values in all analyses as continuous rather than as binary measures of the strength of evidence.

#### Carcass persistence

To investigate how cause of death influenced carcass persistence (i.e., the length of time a carcass provided edible biomass), we fitted a Cox proportional‐hazards model (Cox, 1972). Cox models estimate the hazard rate, or in this case the rate at which carcasses were removed from the environment, while accounting for censored observations. Based on the photographs and field surveys, we defined complete consumption in the context of the typical four stages of ungulate consumption outlined by Wilmers, Stahler, et al. (2003), in which only the brain and hide remain by stage four (~10% of edible biomass). We used stage four to define complete consumption because most scavengers in this system cannot fully process the hide or access the brain. This decision prevented some carcasses from giving the illusion that they persist for very long periods of time when little edible biomass remained. We considered carcasses right censored when they were removed from the view of the camera while edible biomass remained, in which case the carcass was known to have persisted at least until that date. While it would be ideal to measure carcass consumption based on daily biomass removed (rather than as a categorical event), this is difficult to measure from camera footage alone.

The Cox model was fitted in response to (1) a categorical variable for cause of death (wolf, cougar, or roadkill); (2) a continuous variable for mean temperature of the month in which the ungulate was killed (PRISM Climate Group OSU, 2022) to account for seasonal differences in the activity of invertebrates and decomposers; (3) the approximate body mass of the ungulate, based on species, age, and sex; and (4) the proportion of forest cover. We included an interaction between cause of death and temperature to allow for differences in the effect of temperature depending on cause of death. We verified the assumption of proportional hazards using Schoenfeld residuals (functions “ggcoxzph” and “cox.zph” from the “survminer” and “survival” packages; Kassmbara et al., 2021; Therneau et al., 2000).

#### Characterizing community‐wide scavenging

We characterized scavenging activity on roadkill and cougar kills. Because we were prohibited from deploying cameras during the first 1–4 days after abandonment by wolves, when scavengers were likely most active, we did not include wolf kills in the analyses of scavenging activity.

We first sought to characterize general seasonal patterns of scavenging activity as carcasses aged. To do so, we fitted a generalized additive mixed model of summed daily foraging duration of all species on a given carcass (in minutes per day) in response to (1) a factor‐smooth interaction between carcass age (in days), cause of death (cougar, vehicle), and season (summer or winter); (2) the proportion of forest cover; and (3) a random intercept of carcass ID. This structure allowed aggregated, community‐wide scavenging patterns to have different temporal patterns among the causes of death and seasons while accounting for non‐independence of multiple observations at carcasses.

Next, we characterized species‐specific scavenging using summed activity at each carcass for each species (in minutes per carcass). We analyzed these data using joint species distribution models (jSDMs), which provide a model‐based approach for simultaneously modeling the responses of many taxa (Warton et al., 2015). jSDMs have recently become popular for modeling the geographical distributions of many species, but they can also be used to model other properties of community data. jSDMs, and specifically generalized linear latent variable models (GLLVM), are extensions of generalized linear models, but rather than modeling species separately, they introduce unobserved latent predictors to each sample, which induce correlations among taxa (Warton et al., 2015). These latent variables can be thought of as the axes of an ordination, representing the main axes of covariation among species (Warton et al., 2015).

We used the jSDM to infer the effects of cause of death and season on scavenging rates of the major scavengers, while accounting for correlations among species, such as those arising from interactions among species. This model took the form of the scavenging index (in minutes) per carcass modeled in response to categorical variables for cause of death (cougar or roadkill), season (summer or winter), the number of days that a camera was operational (2–31, mean = 27), the proportion of forest cover (0–1), and the approximate carcass weight (based on field‐estimated sex and life stages [neonate, juvenile, yearling, adult]) of each species (range = 5–450 kg, mean = 66 kg).

We fit the jSDMs using the “gllvm” package (Niku et al., 2019). This package fits GLLVMs using a maximum likelihood framework, facilitating use of information criteria to select the number of latent variables that best matches the data. Warton et al. (2015) report that five latent variables is typically more than sufficient. Thus, we fit five models, corresponding to between 1 and 5 latent variables. We used the negative binomial distribution, which fit the data far better than the Poisson distribution (difference in Akaike information criterion between models [ΔAIC] 50,000–87,000), and evaluated models by examining diagnostic plots of residuals.

#### Temporal partitioning of foraging by mammals

We compared temporal patterns of carcass visitation by cougars, coyotes, bobcats, and black bears (insufficient records for wolves) at roadkill and predator kills to evaluate whether animals allocated foraging into periods with lower perceived risk. We expected human activity would be greatest during the day and near roads, with traffic volumes substantially higher during the day than at night (Cunningham et al., 2022). In contrast, we expected cougars and wolves to be primarily crepuscular/nocturnal at their kills (Bassing, 2022; Bassing et al., 2024; Gaynor et al., 2022; Shores et al., 2019). Given these different patterns of expected risk, we considered it reasonable to compare predator kills (cougars and wolves) with roadkill, anticipating greater nocturnality of scavengers at roadkill than predator kills (Gaynor et al., 2018). Animal activity is usually determined by the timing of sunrise and sunset, rather than clock time (Nouvellet et al., 2012). Because our study was conducted across seasons with differing day lengths, we standardized clock time to sun time, with 06:00 corresponding to sunrise and 18:00 corresponding to sunset (“sunTime” function; Ridout & Linkie, 2009). Using independent events, defined as a 30‐min period of independence, we used a non‐parametric kernel density estimate to visualize species activity patterns at roadkill compared with predator kills, and measured the degree of similarly using the coefficient of overlap, where values of 1 indicate perfect overlap and 0 represents no overlap (“overlap” package; Ridout & Linkie, 2009).

#### Hurdle model of carcass visitations by coyotes and bobcats

Durations of carcass visitations (in minutes per day) by coyotes and bobcats were strongly zero‐inflated and did not correspond to usual distributions (e.g., Poisson or negative binomial). Prior studies have inferred that zero‐inflated scavenging data may arise because foraging can be broken into two stages, in which animals must first locate a carcass before deciding how long to spend at it (Cunningham et al., 2018). Thus, we used a hurdle model to break the foraging process into two parts (Potts & Elith, 2006): (1) a binary outcome model for whether a species located a carcass on a given day and (2) a model of non‐zero visit durations, given that an animal did locate a carcass (i.e., the “zero‐hurdle” had been crossed). This approach allows for differentiation in the processes governing carcass discovery and the length of visits.

To fit the first part of the hurdle model, we fitted a generalized linear mixed‐effects model with a binomial distribution for whether a carcass was visited on a given day. Then, for the days on which a carcass was visited, we fitted a linear mixed‐effects model of the log‐transformed duration of daily carcass visits (in minutes per day). Both parts of the hurdle model were fit using the glmmTMB R package (Brooks et al., 2017) in response to four interaction terms that allowed the responses of coyotes and bobcats to differ: (1) scavenger species × cause of death, (2) scavenger species × carcass age, (3) scavenger species × carcass mass, and (4) scavenger species × proportion forest cover. For this analysis, we aggregated wolf and cougar kills into a “predator‐kill” category because of relatively few scavenging events at wolf kills. We accounted for the non‐independence of multiple observations at a carcass using a random intercept of scavenger species nested within carcass ID.

#### Carnivore interactions and vigilance

To investigate the relative risk of a mesocarnivore encountering a large predator at roadkill versus predator kills, we constructed a time‐to‐event model of the elapsed time between visits of mesocarnivores and large predators. We did this by fitting a mixed‐effects Cox proportional‐hazards model, which estimated the risk per unit time of a large carnivore visiting a carcass site after a mesocarnivore did. This procedure required calculating the elapsed time (in hours) between each independent visit (described for temporal partitioning) of a mesocarnivore (coyote or bobcat) and the subsequent observation of a large predator (wolf, cougar, or black bear). If a large predator was not observed following a mesocarnivore, we considered those instances right censored (i.e., the event did not occur within the observation period), in which case we knew only the minimum elapsed time. We aggregated coyotes and bobcats because relatively few bobcat events precluded fitting species‐specific models. Likewise, wolf and cougar kills were aggregated into a “predator‐kill” category because there were relatively few foraging events observed at wolf kills. We fitted the Cox model in response to a (1) categorical variable for cause of death (predator kill or roadkill), (2) continuous variable for carcass age (days since death), (3) continuous variable for carcass weight (in kilograms), and (4) random effect of carcass ID to control for non‐independence of observations on the same camera. We verified the assumption of proportional hazards using Schoenfeld residuals (“cox.zph” function from “survival” package; Therneau, 2022).

Next, we analyzed the use of vigilance by coyotes and bobcats as a strategy to manage risk at kill sites. We defined an animal as vigilant if they had their head up, looking somewhere other than the carcass (Atwood & Gese, 2008). We fitted a generalized linear mixed‐effects model (binomial distribution) of the probability that a coyote or bobcat was vigilant in response to (1) cause of death (predator or vehicle) × scavenger species (coyote or bobcat), (2) carcass age (in days) × scavenger species, and (3) proportion forest cover × scavenger species. We accounted for the non‐independence of repeated measures at each kill site using a random intercept of species nested within carcass ID.

## RESULTS

Wolf and cougar kills generally occurred in areas with low human footprint, whereas roadkill typically occurred in areas with relatively high human footprint (Figure 2C). Of our sample of 139 carcasses, 88% were either mule deer or white‐tailed deer (Table 1). In our non‐systematic sample, 43% of wolf kills were moose, compared with 5% and 0% by cougars and vehicles, respectively (Table 1).

**TABLE 1:** Composition of ungulate carcasses used for the analysis of carcass persistence.

Carcass species	Wolf kill	Cougar kill	Roadkill	Total
Deer	12 (0.57)	57 (0.89)	53 (0.98)	122
Elk	0 (0)	2 (0.03)	1 (0.02)	3
Moose	9 (0.43)	5 (0.08)	0 (0)	14
Total	21	64	54	139

*Note*: The number of each ungulate species is given for each cause of death, with mule and white‐tailed deer aggregated, and proportions in parentheses.

### Carcass persistence

The Cox proportional‐hazards model indicated that carcass persistence times differed significantly among the causes of death (Appendix S1: Table S1). Wolf kills were consumed most rapidly, persisting for a model‐estimated median of 4.7 days, whereas cougar kills and roadkill persisted for a median of 8.9 and 12 days, respectively (Figure 3A). Roadkill was consumed more rapidly with increasing temperature, whereas temperature had no effect on the persistence of wolf or cougar kills (Appendix S1: Table S1). Most wolf kills (14/21) had been completely consumed by the time of the carcass investigations. This pattern suggests that our analysis likely overestimatesd the persistence times of wolf kills and that persistence time differences between the causes of death are likely even larger than estimated here.

### Community‐wide scavenging

Scavenging activity tended to peak several days (summer = 2–3 days, winter = 3–9 days) after the ungulate's death. Scavenging activity was highest and occurred for substantially longer in winter (Figure 3B), when vultures, bears, invertebrates, and decomposers are either less active or inactive.

**FIGURE 4:**
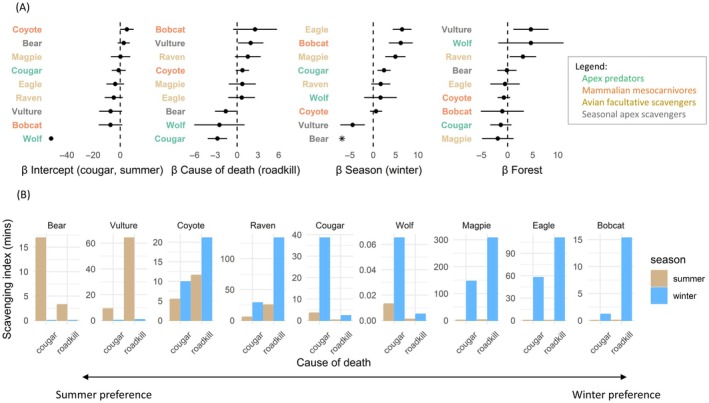
Community‐wide foraging activity differs seasonally and between cougar kills and roadkill. (A) Coefficient estimates (±95% CI) from the joint species distribution model (jSDM). Species are colored according to functional traits. “*” indicates unidentifiable uncertainty bounds on coefficients (because no bears were observed scavenging in winter). (B) Mean scavenging index (in minutes) predicted by the jSDM. Species are roughly ordered from those that scavenge most in summer (left) to those that scavenge most in winter (right). See Appendix S1: Figure S2 for the proportion carcasses visited by each species.

Scavenging differed among the causes of death and seasons. There were strong seasonal effects on carcass visitations, with seasonal apex scavengers—black bears and turkey vultures—dominating carcasses during summer (Figure 4). Most other species, especially bobcats, eagles, and magpies, scavenged much more frequently in winter (Figure 4). Scavenging differed among the causes of death, with large carnivores (cougars, black bears, and wolves) making relatively little use of roadkill, while bobcats, turkey vultures, ravens, and coyotes had a tendency to scavenge more on roadkill than cougar kills (Figure 4A). Increasing forest cover positively influenced scavenging by turkey vultures and ravens, and marginally by wolves (Figure 4A).

### Mitigating risk through temporal partitioning of foraging

**FIGURE 5:**
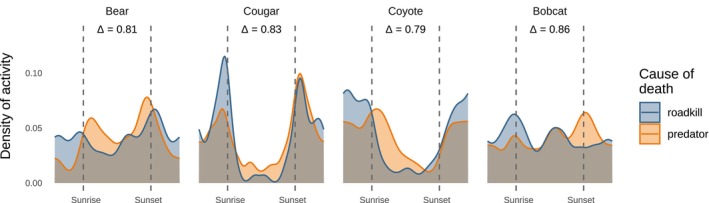
Temporal partitioning of foraging reduces the potential for risky interactions at carcasses. Colors show diel activity patterns at predator kills (orange), which were usually in areas of low human footprint, and roadkill (blue), which were usually in areas of much higher human footprint. Vertical dashed lines show 30 min before sunrise and 30 min after sunset, approximately discriminating darkness from light. “Δ” denotes the coefficient of overlap between a species' activity patterns at predator kills and roadkill.

Cougars had a dominant activity peak in the late afternoon/evening when foraging on their own kills, but this peak shifted to before sunrise when visiting roadkill, consistent with avoidance of predominantly diurnal humans (Figure 5). Black bears, cougars, and coyotes were more nocturnal at roadkill compared with predator kills (Figure 5). Bobcats were equally nocturnal at roadkill and predator kills, but their dominant activity peak shifted from sunset at predator kills to sunrise at roadkill (Figure 5), consistent with subtle avoidance of humans.

### Divergent scavenging strategies of coyotes and bobcats

**FIGURE 6:**
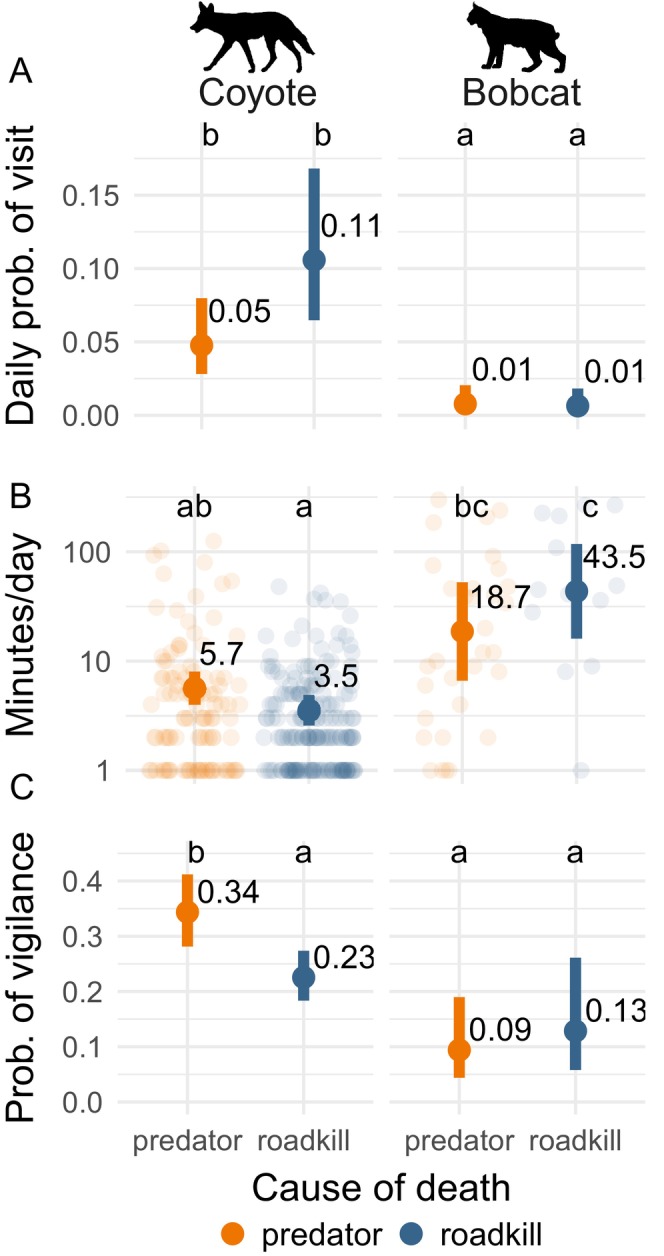
Differing strategies to balance the risks and rewards of scavenging. Coyotes visited carcasses frequently, but usually for short periods of time. By contrast, bobcats infrequently visited carcasses, but usually for relatively long durations. To mitigate the higher risk of encountering a large predator, coyotes increased their vigilance when scavenging on large predator kills. (A) The model‐estimated daily probability that a coyote or bobcat visited a carcass (i.e., part 1 of the hurdle model). (B) The model‐estimated length of visits, conditional on visiting the carcass. The background points show the raw data used in part 2 of the hurdle model. (C) The model‐estimated probability that a coyote or bobcat was vigilant in a given photograph. In all panels, carcass age was held constant at 1 day, error bars show the 95% CI, and letters denote significantly different pairwise comparisons. Silhouettes of coyote by Bob Comix (Creazilla; public domain under a Creative Commons Attribution 4.0 license) and bobcat by Margot Michaud (PhyloPic; public domain under a CC0 1.0 Universal Public Domain Dedication license).

The risk of coyotes and bobcats encountering a large carnivore was substantially higher at predator kills than at roadkill (hazard ratio = 5.6; *p* = 0.001; Appendix S1: Table S2). In the context of this risk, the hurdle model revealed divergent scavenging strategies among the mesocarnivores. The time from an ungulate's death to the first arrival of bobcats and coyotes did not differ significantly (Appendix S1: Figure S3), but the daily probability of visiting a carcass was up to 10‐fold higher for coyotes than for bobcats (Figure 6A). Coyotes visited roadkill 2.4 times more frequently than predator kills, but visits to both were usually brief (model‐estimated mean = 3.5 and 5.6 min, respectively; Figure 6B; Appendix S1: Table S3). By contrast, bobcats infrequently visited roadkill and predator kills (Figure 6A), but when they did, they generally fed for longer durations (model estimate = 41 and 18.3 min, respectively; Figure 6B; Appendix S1: Table S3). Thus, even though bobcats visited carcasses much less frequently, they spent more total time at carcasses than did coyotes (2869 vs. 1966 min in total across all carcasses). When visiting carcasses, coyotes were 1.6 times more vigilant at large predator kills than at roadkill (probability = 0.36 vs. 0.23), whereas bobcats showed no difference in vigilance between predator kills and roadkill (0.1 and 0.14; Figure 6C; Appendix S1: Table S4).

## DISCUSSION

Carnivores play important roles in both provisioning and recycling carrion (Allen et al., 2014; Prugh & Sivy, 2020), and aggressive interactions among carnivores at carcasses may influence scavenging rates, behaviors, and abundance of smaller guild members (Ruprecht et al., 2021; Sivy et al., 2017). In many anthropogenic landscapes, humans kill carnivores at high rates (Darimont et al., 2015) and also provision ecosystems with human‐derived carrion (Newsome et al., 2015). In the multiple‐use landscapes of Washington State, we show that a common anthropogenic carrion subsidy—roadkill—is used widely by the scavenger community, typically more so than predator kills. The rapid removal of wolf kills implies they provide relatively few carrion subsidies in this deer‐dominated system, differing from other well‐studied wolf systems where typical prey are larger (elk and bison), such as Yellowstone National Park (Walker et al., 2018; Wilmers, Crabtree, et al., 2003). Mesocarnivores were at much higher risk of encountering larger predators at predator kills compared with roadkill. Coyotes appeared to mitigate this risk through shorter feeding times and increased vigilance, whereas bobcats visited fewer carcasses but fed for longer, suggesting they use different cues than coyotes to decide when it is safe to scavenge. These patterns highlight the complexity of the risks and rewards associated with scavenging in anthropogenic landscapes, and that scavenging behaviors are jointly influenced by a complex interplay between human impacts and large predators.

Persistence of predator kills differed in predictable ways. Rapid consumption of wolf kills likely resulted from feeding as packs, which ranged in minimum size from 2 to 11 (mean = 5.1; WDFW, 2019). By contrast, cougars are solitary hunters, and they often conceal carcasses in caches (Allen et al., 2023; Knopff et al., 2009). As expected, their kills persisted longer than wolf kills. Relative to the average persistence times of ungulate carcasses from 26 studies (see fig. S1 of Inagaki et al., 2022), persistence of wolf kills (<4.7 days) corresponded to approximately the median persistence time, whereas cougar kills (8.9 days) and roadkill (12 days) corresponded to the upper quartile. There are two main caveats to our characterization of carcass persistence times. First, most wolf kills had been almost entirely consumed by the time of the cluster investigations. Second, the carcasses in our wolf sample are likely biased toward larger carcasses that persist sufficiently long for a GPS cluster to emerge (i.e., moose vs. deer). Collectively, these biases mean wolf kill persistence times are likely overestimated, suggesting the differences between the causes of death are likely even larger than we report. Our findings indicate that wolves likely provision less carrion in ecosystems where small‐bodied ungulates outnumber larger‐bodied ungulates (Walker et al., 2018; Wilmers, Crabtree, et al., 2003). Indeed, wolf reintroduction to Yellowstone shifted carrion inputs from a late‐winter pulse to a steady stream from wolf kills (Walker et al., 2018; Wilmers, Crabtree, et al., 2003), and European bison (*Bison bonasus*, ~500 kg) in Poland provided carrion for an average of 106 days per carcass (Selva et al., 2003). The ungulate community at our study sites, however, is mostly composed of smaller deer species (<70 kg) that, when coupled with the rapid consumption of wolf kills observed here, suggests wolves provision other species with relatively little carrion in this system. Given wolves themselves are facultative scavengers, it remains an open question whether their overall effect is to increase or decrease carrion available to other scavengers. The answer would likely depend on whether the growth rate of the prey population responds to wolf predation (Wilmers & Getz, 2004), the extent to which wolves monopolize carrion from sources other than their own kills, and the proportion of their kills that become available to other species. This study provides insights into the latter two points, showing that wolves infrequently visited cougar and roadkill carcasses compared with other scavengers (Figure 4) and that they left only a small proportion of their kills for other species.

The community‐wide differences in scavenging across causes of death and seasons shed light on risk‐sensitive interactions among animals. Large carnivores (wolves, cougars, and black bears) foraged more on cougar kills than roadkill, consistent with avoidance of humans, their greatest threat in this system (Prugh et al., 2023). Conversely, avian scavengers and mesocarnivores made greater use of roadkill, highlighting their role in recycling anthropogenic carrion. The distinct seasonal pattern of scavenging, where turkey vultures and black bears dominate during summer, underscores the profound impact of apex scavengers (O'Bryan et al., 2019). These findings are novel because few studies have been able to compare large predator and anthropogenic sources of carrion (but see Wilmers, Stahler, et al., 2003), presumably because large predators mostly occur in landscapes with relatively low human influence (Ripple et al., 2014). Ideally, we would have also analyzed community scavenging on wolf kills, but their rapid consumption and our delay in visiting them (dictated by management regulations), combined with a deer‐dominated system, meant that carcasses were frequently entirely consumed by the time of the carcass investigations, and consequently, few scavenging events were observed on wolf kills.

Roadkill occurred in areas of relatively high human footprint (Figure 1; Appendix S1: Figure S1) and hosted mostly avian scavengers and mesocarnivores (Figure 4). Unsurprisingly, roadkill persisted for the longest durations, likely due to the absence of larger predators at the time of the ungulate's death. Roadkill was consumed more rapidly during warmer months (Appendix S1: Table S1), when microbes, invertebrates, bears, and vultures were active. Of these factors, vultures may be most influential, with other research in Spain and Argentina showing their importance in accelerating carcass consumption (Sebastián‐González et al., 2013). Likewise, exclusion experiments reveal large increases in persistence times of carcasses without vultures in Kenya, Spain, and the United States (Hill et al., 2018; Morales‐Reyes et al., 2017; Ogada et al., 2012), demonstrating the important ecosystem service provided by obligate scavengers, especially given their willingness to scavenge on human‐derived carrion.

The mammalian mesocarnivores—coyotes and bobcats—exhibited divergent scavenging strategies, demonstrating that approaches to scavenging and risk mitigation can be context dependent and hard to generalize, even within the same guild. Coyotes visited carcasses frequently, but often for short durations and with increased vigilance on predator kills. By contrast, bobcats infrequently scavenged and did not reveal any differential use of vigilance as a risk‐mediation strategy. Despite infrequent visits, bobcats had the longest foraging bouts, suggesting they use different cues than coyotes to decide when a carcass provides suitably safe foraging opportunities. One possibility is that these differences in carcass visitations arise from species‐specific sensory capabilities: coyotes' superior sense of smell and coursing locomotion likely aid in carcass detection, resulting in higher visitation rates. Bobcats, with lesser olfactory abilities and a stalking hunting mode, may detect fewer carcasses, but their longer foraging bouts suggest that once a sufficiently safe carcass is located, they may exploit it more fully. Another possible explanation may involve feeding characteristics: coyotes frequently dragged away small parts of carcasses for consumption or caching elsewhere, whereas bobcats rarely did so. Our finding that bobcats engage in long foraging bouts contrasts with the finding of Ruprecht et al. (2021), who reported that bobcats in Oregon, USA, did not visit any cougar‐killed carcasses and their scats contained little evidence of scavenging on large mammals. Our results, however, agree that coyotes are far more likely to visit carcasses than bobcats—between 4 and 10 times in our study system (Figure 6A). The higher frequency of carcass visits by coyotes than by bobcats indicates that coyotes would likely interact more frequently with larger predators, suggesting coyote populations are more prone to being influenced by negative interactions at carcasses. This prediction matches the pattern of mesopredator mortality in this system, whereby large carnivores (mainly cougars) caused an estimated annual mortality of 10.5% for collared coyotes and 6.5% for bobcats (Prugh et al., 2023).

Most studies of the effects of large carnivores have been conducted in protected areas, where humans cause relatively little wildlife mortality. Outside of protected areas, humans are uniquely lethal predators (Darimont et al., 2015), and in our multiple‐use system, humans kill bobcats and coyotes at more than three times the rate of large predators (Prugh et al., 2023). The spatiotemporal risk landscape is thus more complex than in protected areas because humans squeeze apex predators into fragments of time and space (Palmer et al., 2023; Prugh et al., 2023). In our study, the temporal patterns of foraging by cougars, bears, coyotes, and bobcats showed clear recognition of the competing risks posed by humans and other predators. Visits to roadkill preferentially occurred at night, presumably reflecting the higher risk of encountering humans near roads during the day. By contrast, visits to predator kills were more diurnal, seemingly also responding to the higher risk of encountering large carnivores during crepuscular and nocturnal periods (Bassing, 2022; Bassing et al., 2024; Gaynor et al., 2022; Shores et al., 2019). Our findings agree with earlier camera research in eastern Washington showing that coyotes were more diurnal/crepuscular in areas with wolves, and less diurnal in areas without wolves (Shores et al., 2019). Collectively, this flexibility in temporal activity indicates a fine‐tuned understanding of the competing risks posed by wolves and humans and the respective periods in which those risks are likely highest.

Landscapes heavily impacted by humans are increasingly important for conserving large carnivores as well as the species they influence jointly with humans (Oriol‐Cotterill et al., 2015). In our system, humans strongly influenced the distribution of carrion on the landscape (Figure 2C), and therefore the distribution of costs and benefits. Avoidance of humans by wolves and cougars in this system (Prugh et al., 2023) restricted their carrion provisioning to less‐impacted pockets of the landscape (Figure 2C). By implication, this provides areas where smaller scavengers can potentially access carrion with little risk from large predators, so long as they can navigate anthropogenic pressures. Roadkill in particular raises the concern of generating an ecological trap in which carnivores are attracted to roadside carcasses, maladaptively increasing their own risk of death. Ecological traps in this regard are likely most pronounced for species that are less agile or have life‐history traits, such as slow reproduction, that increase susceptibility to the fitness cost of mortality (Battin, 2004). Indeed, it has been suggested that roadkill provides an ecological trap for golden eagles in Scandinavia (Singh et al., 2024) and Tasmanian devils (*Sarcophilus harrisii*) and eastern quolls (*Dasyurus viverrinus*) in Australia (Jones, 2000). However, bobcats and coyotes in this study system were rarely killed by vehicles (1 of 72 collared animals; Prugh et al., 2023), suggesting foraging on roadkill is likely a net benefit to them. They are, however, more susceptible to being shot or trapped (24 of 72) (Prugh et al., 2023), and it remains an open question whether attraction to roadkill inadvertently places them at higher risk of other threats, especially those to which these species may be evolutionarily naïve, like guns (Darimont & Shukla, 2023; Prugh et al., 2023). Furthermore, if the benefits do exceed the costs, it remains an open question whether these anthropogenic subsidies inflate scavenger populations and have downstream suppressive effects on other species and carrion persistence.

If large predators continue to reclaim anthropogenic landscapes around the world (Chapron et al., 2014; Ingeman et al., 2022; Ripple et al., 2022), they will add complexity to the spatiotemporal landscape of risks and rewards associated with scavenging. Our results in the context of others (Selva et al., 2003; Walker et al., 2018; Wilmers, Crabtree, et al., 2003) indicate that the extent of carrion provisioning by predators depends on the relative body sizes of the prey community, with ecosystems dominated by larger‐bodied prey seemingly providing more leftovers for other scavengers. Humans strongly influence scavenging, both directly by providing sources of carrion for scavengers (e.g., roadkill) and indirectly by restricting the locations of predator‐derived carrion. Hence, outcomes of predator recovery, and for scavenger guilds more broadly, will depend on the composition of the prey community, the speed at which large predators consume their kills, and the relative vulnerability of scavengers to the competing risks posed by humans and large predators.

## AUTHOR CONTRIBUTIONS

*Conceptualization*: Laura R. Prugh and Calum X. Cunningham. *Field methodology*: Laura R. Prugh, Rebecca Windell, Taylor R. Ganz, Lauren C. Satterfield, and Aaron J. Wirsing. *Data preparation*: Rebecca Windell, Lauren C. Satterfield, and Calum X. Cunningham. *Analysis and visualization*: Calum X. Cunningham. *Writing—original draft*: Calum X. Cunningham. *Writing—review and editing*: all authors.

## CONFLICT OF INTEREST STATEMENT

The authors declare no conflicts of interest.

## Supporting information


Appendix S1:

## Data Availability

Data and code (Cunningham et al., 2025) are available in Figshare at https://doi.org/10.6084/m9.figshare.28537280.v1.
