# Supplementary material for: Navigating the risks and rewards of scavenging in multipredator, human‐impacted landscapes
Source: Ecology. 2025 May 8;106(5):e70090. doi: 10.1002/ecy.70090 (PMC12060637; doi:10.1002/ecy.70090)
Supplement: Supplementary file 1 — Appendix S1: [file ECY-106-e70090-s001.pdf]

Appendix S1.

Navigating the risks and rewards of scavenging in multipredator, human-impacted landscapes

Ecology

Calum X. Cunningham, Rebecca Windell, Lauren C. Satterfield, Aaron J. Wirsing, Thomas M. Newsome, Taylor R. Ganz, Laura R. Prugh

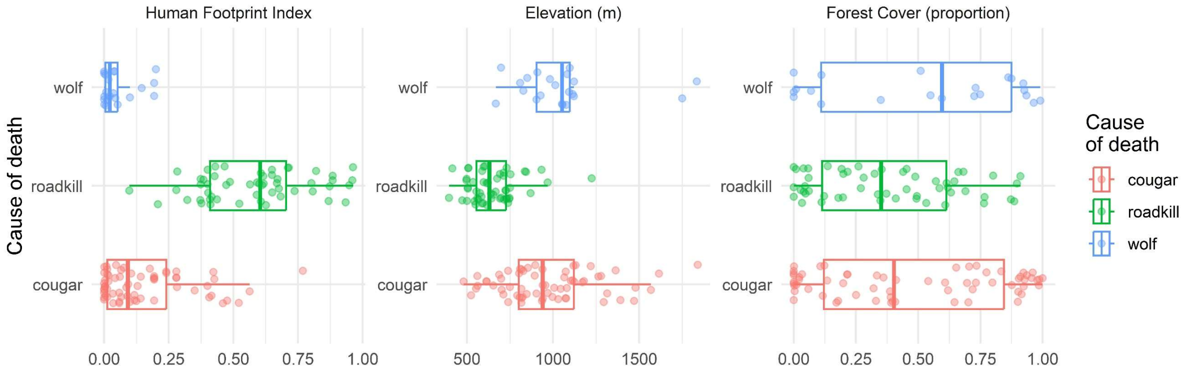

Figure S1. Associations between cause of death and potential explanatory variables. Cause of death was strongly associated with human footprint index and elevation. This pattern likely occurs because humans, who preferentially occupy lower elevations, play a large role in shaping large predator distributions, rendering human footprint and elevation unsuitable for inclusion in models that include cause of death. The percentage of forest cover was uncorrelated with cause of death, and we therefore included it as a covariate to control for its potential effect on carcass persistence and scavenging behaviors.

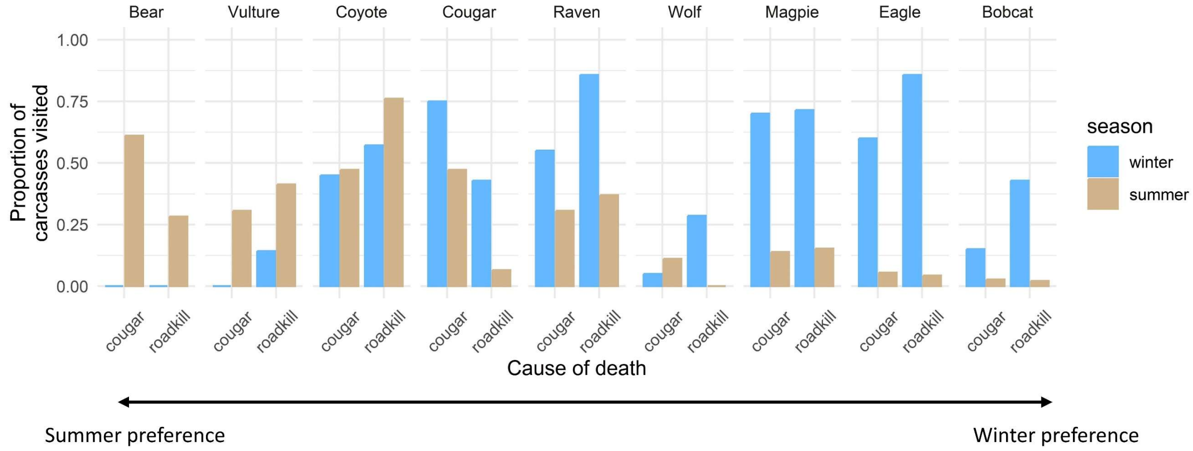

Figure S2. Proportion of carcasses visited by each species. Species are roughly ordered from those that scavenge most in summer (left) to those that scavenge most in winter (right).

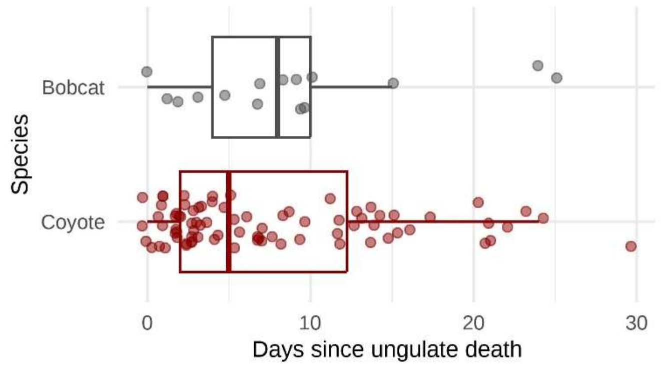

Figure S3. Equivalent first arrival time to a carcass by bobcats and coyotes. The graph shows the number of days between the ungulate's death and the first arrival of a bobcat and a coyote on a carcass. First arrival did not differ significantly between bobcats and coyotes (Welch t-test: $p = 0.496$, $t = 0.694$, $df = 18.86$).

Table S1. Results from a Cox proportional-hazards model of carcass persistence times. "COD" refers to cause of death, relative to the baseline category of cougar kills. "Temp" refers to the mean temperature of the month in which the ungulate was killed. Coefficient estimates show a variable's model-estimated effect on the log-hazard rate, and exp(coef) shows its effect on the hazard rate.

Variable	coef	exp(coef)	se(coef)	z	Pr(>|z|)	
CODroadkill	-1.165	0.312	0.658	-1.771	0.077	.
CODwolf	1.391	4.019	0.361	3.852	<0.001	***
Carcass weight	-0.005	0.995	0.002	-2.701	0.007	**
Temp	0.007	1.007	0.020	0.351	0.725	
Forest	-0.263	0.769	0.423	-0.621	0.534	
CODroadkill × Temp	0.073	1.076	0.041	1.778	0.075	.
CODwolf × Temp	-0.041	0.960	0.040	-1.032	0.302	

Table S2. Results from a Cox proportional-hazards model of the time between mesopredator and large predator observations. "COD" refers to cause of death, relative to the baseline category of roadkill. Coefficient estimates show a variable's model-estimated effect on the log-hazard rate, and exp(coef) shows its effect on the hazard rate.

Variable	coef	exp(coef)	se	p	
CODpredator	1.722	5.597	0.548	0.001	**
Carcass age	-0.058	0.943	0.022	0.009	**
Carcass weight	-0.0004	1.000	0.003	0.912	

Table S3. Results from the hurdle model of carcass visitations and foraging times. "COD" refers to cause of death, relative to the baseline category of predator kill. Species refers to the scavenger species, relative to the baseline category of coyote.

	Part 1: carcass visitations				Part 2: foraging time			
Variable	Coef	SE	P		Coef	SE	P	
Intercept	-2.523	0.360	<0.001	***	1.624	0.221	<0.001	***
CODroadkill	0.860	0.360	0.017	*	-0.473	0.214	0.027	*
SpeciesBobcat	-2.577	0.744	0.001	***	1.458	0.691	0.035	*
Carcass age	-0.047	0.008	<0.001	***	-0.030	0.008	<0.001	***
Carcass weight	0.108	0.177	0.540		-0.028	0.131	0.828	
Forest	-0.981	0.529	0.064	.	0.341	0.327	0.296	
CODroadkill:SpeciesBobcat	-1.053	0.734	0.151		1.319	0.713	0.065	.
SpeciesBobcat:carcassAge	-0.068	0.028	0.015	*	-0.062	0.025	0.014	*
SpeciesBobcat:carcass weight	0.664	0.262	0.011	*	0.101	0.247	0.683	
SpeciesBobcat:forest	1.820	1.004	0.070	.	-0.499	1.020	0.625	

Table S4. Results from the GLMM of vigilance. "COD" refers to cause of death, relative to the baseline category of roadkill. Species refers to the scavenger species, relative to the baseline category of coyote.

Variable	Estimate	SE	P	
Intercept	-0.683	0.178	<0.001	***
Carcass age	-0.024	0.004	<0.001	***
SpeciesBobcat	-1.699	0.540	0.002	**
CODpredator	-0.588	0.180	0.001	**
Forest	0.306	0.272	0.260	
carcassAge:SpeciesBobcat	0.097	0.014	<0.001	***
SpeciesBobcat:CODpredator	0.939	0.508	0.064	.
SpeciesBobcat:forest	-0.082	0.719	0.910	
